# Stable Elemental Fingerprints for Multi-Attribute Classification of Ginseng Using Laser-Induced Breakdown Spectroscopy

**DOI:** 10.3390/plants15203124

**Published:** 2026-10-13

**Authors:** Yujia Dai, Yilei Zhang, Huangwei Li, Shangyong Zhao, Ziyuan Liu

**Affiliations:** College of Optical, Mechanical and Electrical Engineering (College of Intelligent Equipment Engineering), Zhejiang A&F University, Hangzhou 311300, China

**Keywords:** ginseng, laser-induced breakdown spectroscopy, proximal sensing, nested cross-validation, filter-based wavelength selection, standard normal variate, technical replicates, medicinal plants

## Abstract

Laser-induced breakdown spectroscopy (LIBS) was evaluated for qualitative within-batch discrimination of ginseng materials by plant category, geographical origin, and growth year. The datasets comprised 200 spectra from four plant categories, 100 spectra from two origins, and 100 spectra from two growth-year groups; each class contained 50 pellets prepared from one ground-and-sieved powder batch. Four chemometric pipelines were compared using five-fold nested cross-validation repeated five times, with preprocessing, wavelength filtering, and tuning restricted to the training folds. The best mean balanced accuracies were 1.000, 0.982, and 1.000 for plant category, origin, and growth year, respectively. An SNV-ANOVA50 filter pipeline retained balanced accuracies of 1.000, 0.980, and 1.000 with 50 of 12,990 detector channels. Conservative wavelength matching, limited to the nominal 0.09 nm instrumental resolution, yielded tentative Ba, Al, Ca, and Fe candidates; these matches do not establish elemental identity. A full-spectrum plus standard normal variate (SNV) control showed that resistance to multiplicative scaling and additive offsets arose primarily from SNV rather than from wavelength selection. Detector-channel shifts remained damaging, especially for the filtered pipeline. Because class identity is confounded with powder-batch identity, the reported cross-validation quantifies technical pellet-level separability within the measured materials and does not estimate performance across independent biological batches.

## 1. Introduction

Ginseng is a high-value medicinal plant for which botanical identity, geographical origin, and growth duration influence quality assessment, commercial grading, and traceability. Processing into slices or powders weakens visible diagnostic traits, while conventional chromatographic and molecular assays can require extensive preparation, reagents, or targeted prior knowledge. Spectral methods offer a complementary route because they can capture chemical variation rapidly and couple it with multivariate classification. In ginseng, LIBS and hyperspectral imaging have already been used to distinguish plant categories, regions, and ages [1], and recent studies have extended this direction through LIBS-hyperspectral fusion [2], near-infrared-LIBS fusion [3], and LIBS-Raman fusion [4]. Collectively, these studies show that elemental and molecular spectra contain useful provenance information, but they also raise the practical question of how much discrimination can be retained with a compact LIBS fingerprint.

Laser-induced breakdown spectroscopy (LIBS) focuses a pulsed laser onto the sample surface, forms a transient plasma, and records element-dependent atomic and ionic emission. The technique provides rapid multielement measurement with limited chemical preparation and has been applied to food authentication and quality assessment [5]. Reviews of plant materials describe direct analysis of leaves, roots, fruits, wood, and pressed plant powders [6], while broader agricultural reviews cover nutrient diagnosis, soil and crop monitoring, contaminants, and product classification [7,8,9,10,11]. These studies consistently identify matrix effects, ablation variability, spectral redundancy, and calibration transfer as barriers between high laboratory accuracy and dependable analytical deployment.

Medicinal plants are a particularly relevant application because species substitution, origin mislabeling, and compositional heterogeneity can alter both value and use. LIBS combined with chemometrics has differentiated medicinal plant tissues and elemental profiles [12], and PCA-neural-network models have classified Chinese herbal medicines by material type, origin, or plant part [13]. Plant-leaf classification using selected LIBS lines has further shown that compact elemental variables can retain high discrimination [14]. Elemental fingerprints have also supported geographical classification of tea [15], while combined LIBS and hyperspectral data have been used to trace rice origin [16]. These examples place ginseng within a broader movement toward rapid proximal spectroscopy for plant-resource authentication.

Recent agricultural spectroscopy studies illustrate complementary aspects of this development. A deep spectral autoencoder was used for non-targeted hyperspectral screening of Fritillaria adulterants [17], and hyperspectral models were developed for sodium saccharin quantification in cat food powder [18], nested-cross-validated provenance classification of Curcumae Radix [19], and sulfite analysis in goji berry powder [20]. A controlled bean-seed benchmark published in the target Plants Special Issue similarly compared machine- and deep-learning strategies across visible, near-infrared, and shortwave-infrared spectra [21]. Although these studies use different matrices and spectral mechanisms, they demonstrate the importance of preprocessing, validation design, feature representation, and explicit robustness testing in agricultural spectroscopy.

The same methodological issues are prominent in LIBS. Discriminative restricted Boltzmann machines have been proposed for classification under high-dimensional, small-sample conditions [22]; uncertainty-aware neural networks have been used for small-sample LIBS quantification [23]; and self-supervised blind-spot denoising with Kolmogorov-Arnold networks has addressed spectral noise and nonlinear calibration [24]. Such models can extract complex structure, but higher model complexity does not remove the need for leakage-free validation or stable chemical interpretation. For datasets with many wavelength channels and comparatively few biological materials, the reliability of the validation hierarchy is at least as important as nominal model flexibility.

A further issue is whether a classifier depends on broad intensity patterns or on a small set of reproducible wavelength regions. In this study, the term wavelength-selected pipeline denotes a filter method: SNV preprocessing is followed by univariate ANOVA ranking, retention of a fixed number of channels, and an ordinary linear SVM. It is not an embedded sparse estimator such as an L1-regularized or elastic-net model. A full-spectrum plus SNV control is therefore required to distinguish the effect of normalization from the effect of wavelength filtering in perturbation tests.

This study evaluated LIBS measurements acquired in a separate experiment using the same sample-class design and acquisition settings as earlier ginseng studies [1,2]. The spectra analyzed here were not taken from those reports, and no hyperspectral, near-infrared, or Raman variables were used. The methodological contribution is a leakage-controlled comparison of four classifiers, repeated wavelength-filter stability analysis, compression across several channel budgets, and computational stress testing with an explicit full-spectrum SNV control. The study asks how much technical discrimination is retained after wavelength filtering and which acquisition perturbations threaten that discrimination within the present batch-limited design.

## 2. Results

### 2.1. Spectral Patterns and Exploratory Structure

**Figure 1:**
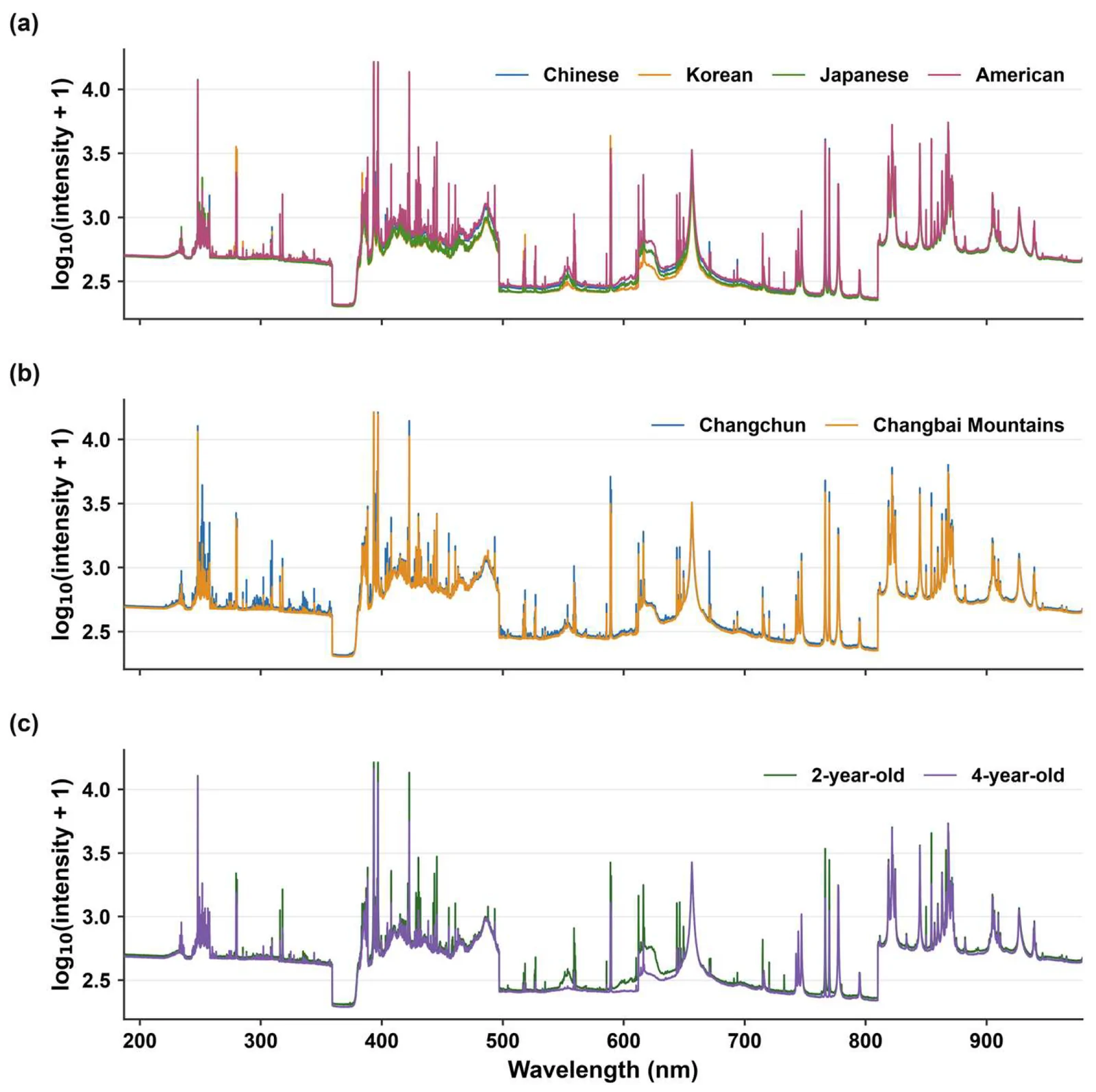
Mean log10-transformed LIBS spectra for (**a**) four plant categories, (**b**) two geographical origins, and (**c**) two growth years. Solid curves show class means and shaded bands show ±1 standard deviation across the 50 pellet-level averaged spectra in each class. AU, arbitrary units.

The wavelength vector covered 186.873–979.230 nm. The class-mean spectra shared a common plant-matrix emission background but showed task-dependent differences in peak intensity (Figure 1). The strongest between-class contrasts were concentrated in narrow wavelength regions rather than appearing as uniform displacement of the full spectrum. This pattern supports evaluating both global multivariate models and targeted wavelength-selection strategies.

Across the three datasets, the main emission structures occurred at comparable wavelengths, as expected for plant powders with broadly similar matrices. Class differences were expressed primarily as changes in relative peak magnitude and in groups of neighboring channels. The plant-category spectra showed the broadest visible deviations, whereas the two origin means were more similar over much of the range and diverged at selected peaks. The two growth-year classes showed repeated intensity differences in several visible and near-infrared regions. The logarithmic transformation was used only for display. Because plasma temperature, electron density, local thermodynamic equilibrium, self-absorption, and wavelength-dependent detector response were not determined, these spectra are interpreted as qualitative multivariate emission signatures rather than elemental concentrations [25].

**Figure 2:**
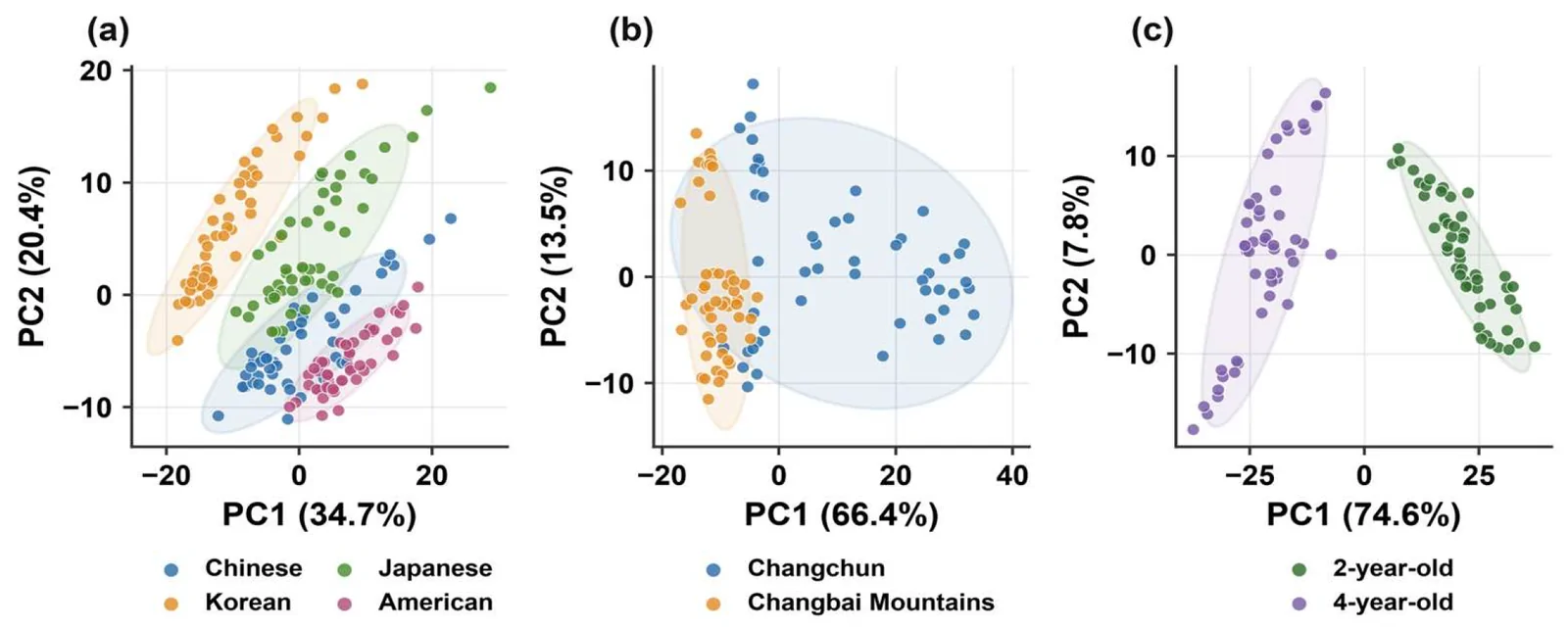
Principal component analysis (PCA) score plots after standard normal variate preprocessing for (**a**) plant category, (**b**) geographical origin, and (**c**) growth year. Ellipses represent covariance dispersion at two standard deviations. Percentages indicate variance explained by the first two principal components.

The first two principal components explained 55.1% of the variance for plant category, 79.9% for geographical origin, and 82.4% for growth year (Figure 2). Separation in the displayed score space was strongest for growth year and geographical origin. The four plant categories showed clear structure but greater overlap in two dimensions, indicating that additional components were useful for supervised classification.

For plant category, the Korean and American groups occupied largely different regions of the PC1-PC2 plane, while the Chinese and Japanese groups were closer and showed partial overlap. This structure explains why the first two components provided useful visualization without fully representing the decision space used by the supervised models. In the origin dataset, the Changbai Mountains group was concentrated mainly toward negative PC1 scores, whereas the Changchun group extended across a wider score range. The growth-year classes were separated principally along PC1, with only limited contact between their covariance ellipses. Thus, the exploratory analysis suggested that growth year was the most strongly expressed low-dimensional contrast, while plant category required information beyond the first two components.

### 2.2. Classification Performance

**Figure 3:**
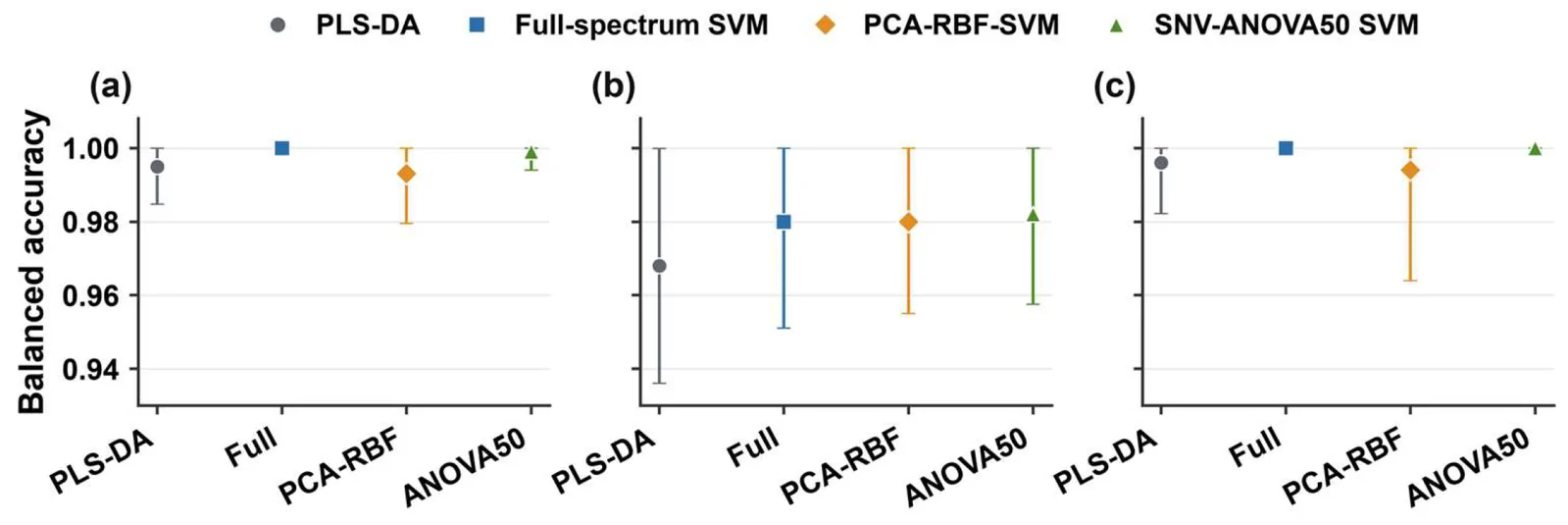
Balanced accuracy of four classification pipelines for (**a**) plant category, (**b**) geographical origin, and (**c**) growth year. Symbols show means and error bars show standard deviations across 25 repeated outer-fold evaluations. ANOVA50 denotes univariate filter selection of the top 50 channels; PLS-DA, partial least-squares discriminant analysis; PCA, principal component analysis; RBF, radial basis function; SNV, standard normal variate; SVM, support vector machine.

All four pipelines separated the controlled pellet spectra accurately (Table 1; Figure 3). Plant-category balanced accuracy ranged from 0.993 to 1.000, whereas growth-year balanced accuracy ranged from 0.994 to 1.000. Geographical origin was the most difficult task, with balanced accuracy ranging from 0.968 for PLS-DA to 0.982 for the SNV-ANOVA50 SVM. These estimates describe outer-fold evaluations of technical pellet replicates from the measured powder batches; they are not independent-batch validation results.

For plant category, the full-spectrum linear SVM achieved 1.000 balanced accuracy in every outer fold. PLS-DA, PCA-RBF-SVM, and the SNV-ANOVA50 SVM also exceeded 0.99 on average, with standard deviations of 0.010, 0.014, and 0.005, respectively. Agreement among balanced accuracy, macro-F1, and MCC shows that no one category dominated the numerical results. The low outer-fold variation of the filtered pipeline indicates consistent pellet-level separation within this batch structure, but it does not demonstrate stability across biological batches.

Origin classification showed a different pattern. The best mean balanced accuracy was 0.982 for the SNV-ANOVA50 SVM, followed by 0.980 for both the full-spectrum linear SVM and PCA-RBF-SVM; PLS-DA reached 0.968. Standard deviations of 0.024–0.032 were larger than those observed for the other tasks. These results identify origin as the least stable within-batch classification problem. For growth year, the full-spectrum and SNV-ANOVA50 linear SVMs both achieved 1.000, while the two latent-variable approaches remained close to unity but showed occasional outer-fold errors.

### 2.3. Spectral Compression

**Figure 4:**
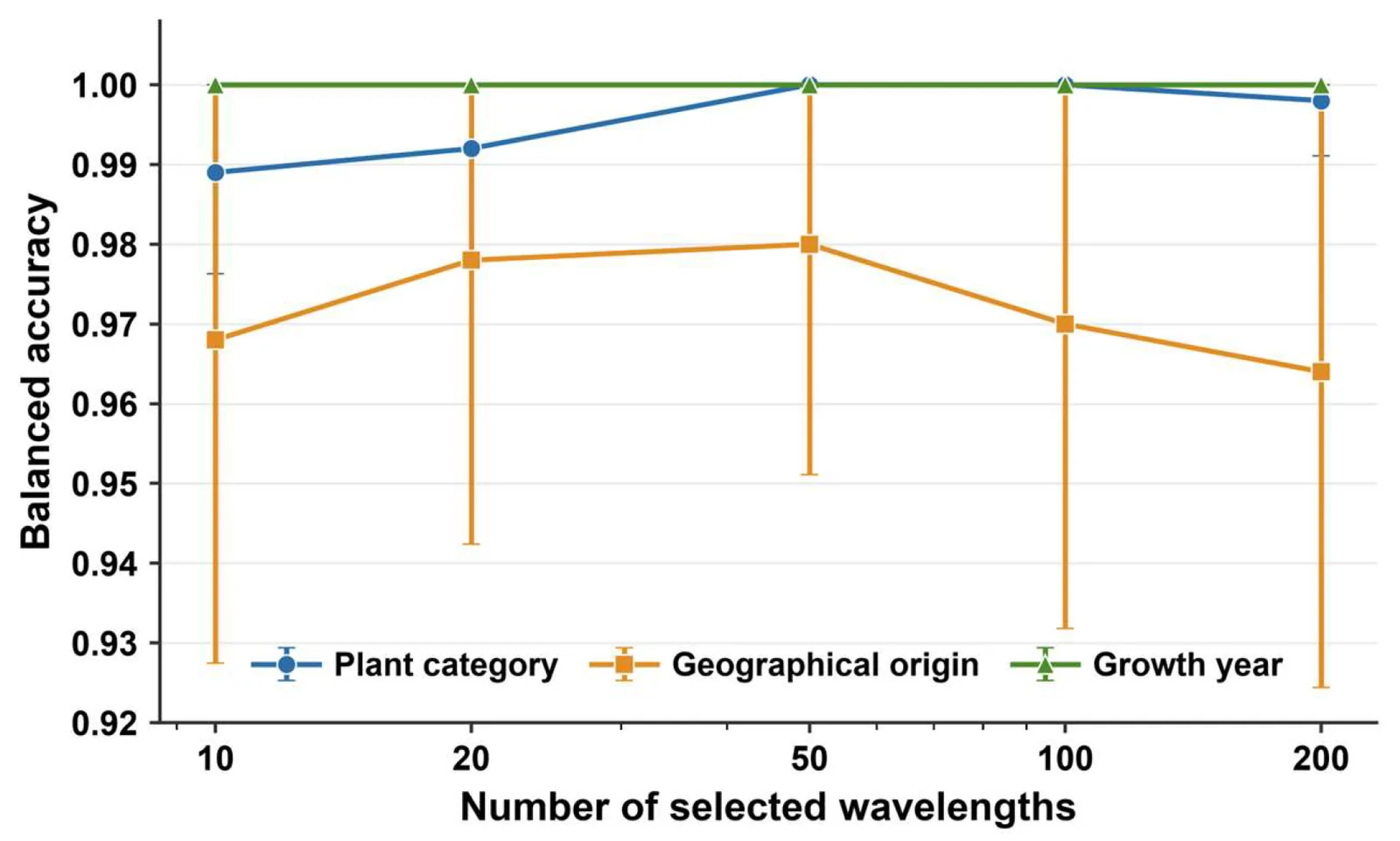
Balanced accuracy after retaining 10–200 ANOVA-ranked wavelengths. Points show means, and error bars show standard deviations across 25 repeated outer-fold evaluations.

Performance as a function of wavelength budget is summarized in Figure 4. Ten wavelengths yielded mean balanced accuracies of 0.989, 0.968, and 1.000 for plant category, geographical origin, and growth year, respectively. Twenty wavelengths already produced 0.978 for origin, compared with 0.980 at 50 wavelengths. The 50-channel setting is therefore used as a common reference budget for feature-stability and perturbation analyses, not as a statistically proven or task-specific optimum.

The compression trajectories differed by task (Figure 4). Plant-category performance increased from 0.989 with 10 wavelengths to 1.000 with 50 and 100 wavelengths. Growth-year performance remained at 1.000 throughout the tested range. Origin accuracy changed only slightly from 0.978 at 20 wavelengths to 0.980 at 50 wavelengths and then decreased to 0.970 and 0.964 at 100 and 200 wavelengths. Given the overlapping outer-fold variability, the 0.002 difference between 20 and 50 wavelengths should not be interpreted as evidence that 50 is uniquely optimal.

The absence of monotonic improvement shows that larger channel sets were not consistently advantageous. Fifty channels were retained only as a common engineering benchmark: it was the smallest tested budget reaching the plant-category maximum, preserved the growth-year maximum, and remained close to the best origin result while reducing input dimension by 99.62%. Task-specific deployment could reasonably use fewer channels after validation on independent batches.

### 2.4. Stable Wavelengths and Candidate Elemental Interpretation

**Figure 5:**
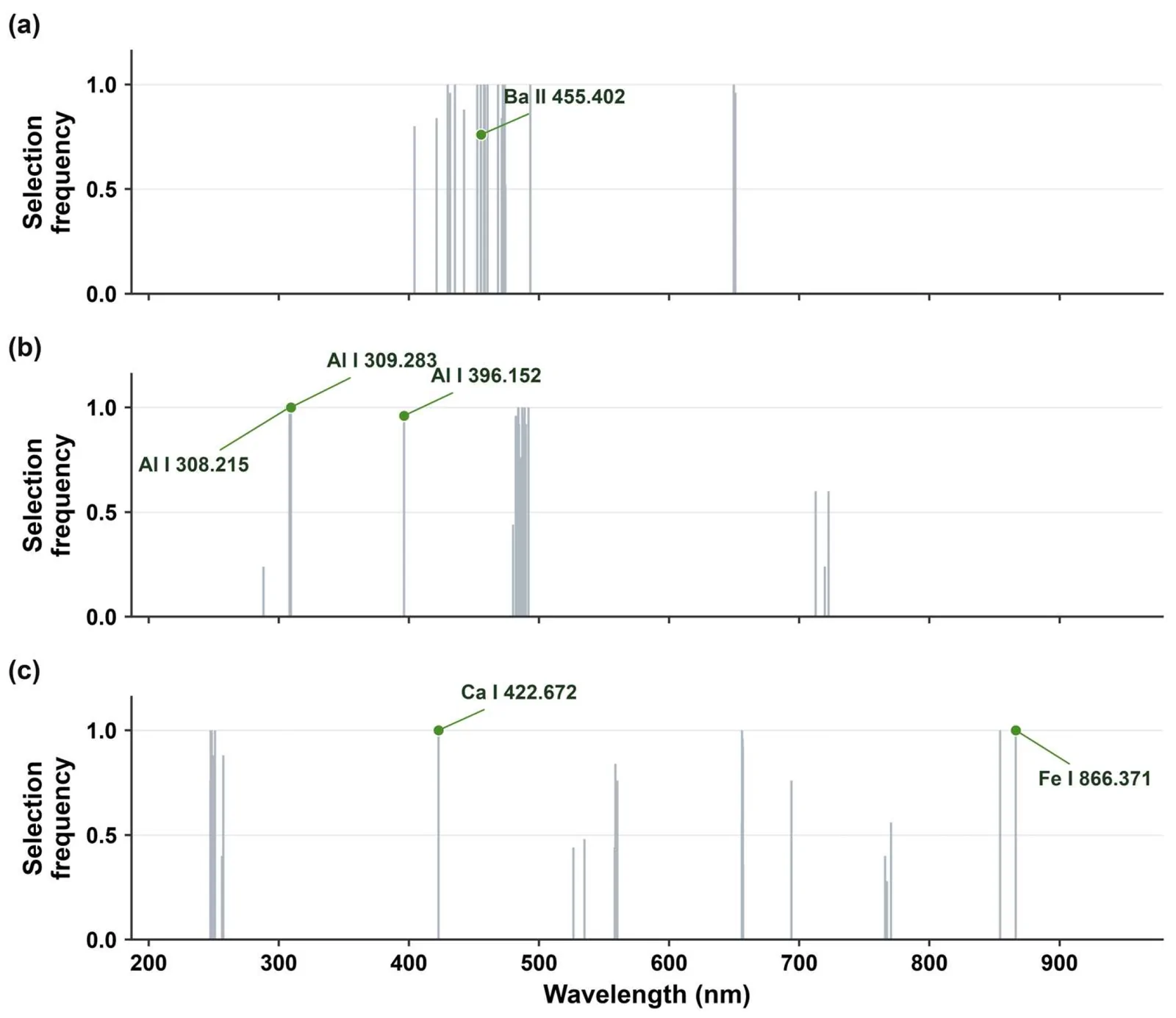
Selection frequency of ANOVA-ranked wavelength variables across 25 outer training folds for (**a**) plant category, (**b**) geographical origin, and (**c**) growth year. Gray lines mark selected channels, and green points identify tentative candidate lines within 0.09 nm. Proximity does not establish elemental identity.

The filter-selected models repeatedly identified compact wavelength regions that differed among tasks (Table 2; Figure 5). After candidate matching was restricted to an absolute difference of 0.09 nm, the nominal instrumental resolution, tentative matches remained for Ba II near 455.402 nm in the plant-category task, Al I near 308.215, 309.283, and 396.152 nm in the origin task, and Ca I 422.672 nm and Fe I 866.371 nm in the growth-year task. These labels indicate only nearby database lines and do not establish that the corresponding elements caused class separation.

Selection frequency distinguished isolated channels from regions repeatedly recovered during the 25 outer training folds. The tentative Ba II match occurred in 76% of folds, the three tentative Al I matches occurred in 96–100%, and the tentative Ca I and Fe I matches occurred in every fold. Previously discussed matches at 493.282, 431.720, 404.314, and 854.379 nm were removed because their offsets from candidate reference lines exceeded 0.09 nm. They are now treated as unassigned wavelength regions.

The unassigned channels are important for interpreting the filter output. Several clusters contained adjacent detector positions with high selection frequency, consistent with broadened features, blends, or correlated background rather than independent chemical markers at every channel. The selected variables should therefore be treated as wavelength neighborhoods. Elemental identity and concentration differences require calibration standards, local line-shape analysis, and an independent method such as ICP-MS or ICP-OES.

### 2.5. Sensitivity to Spectral Perturbations

**Figure 6:**
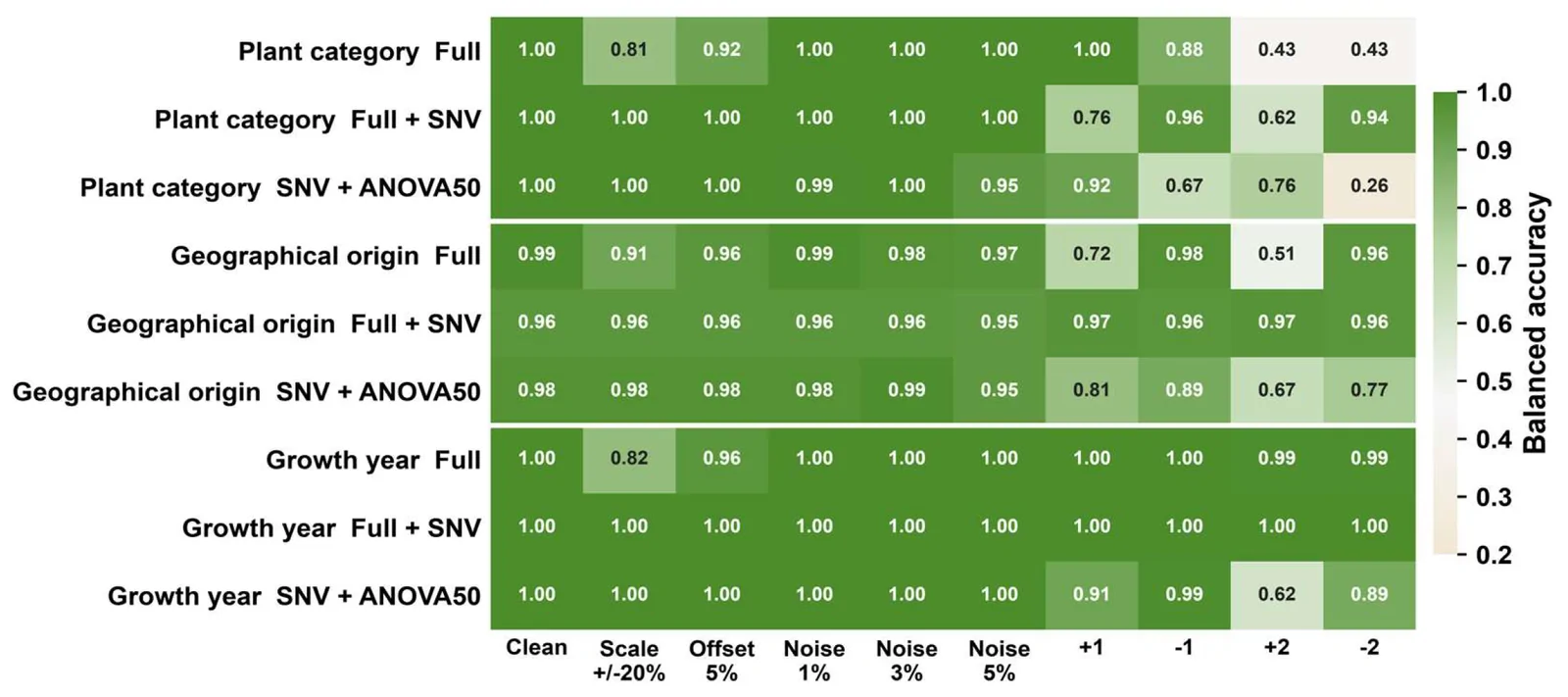
Absolute balanced accuracy under perturbations applied only to held-out spectra. The three pipelines separate the effects of SNV and ANOVA50 wavelength filtering. Columns show perturbation conditions; rows show task-pipeline combinations. Shift values denote detector indices. SVM, support vector machine.

Gaussian noise up to 5% produced limited changes in all three linear-SVM pipelines (Figure 6). Under spectrum-specific scaling factors drawn uniformly from 0.8 to 1.2, balanced accuracies for the full-spectrum SVM were 0.81, 0.91, 0.82, whereas the full-spectrum plus SNV control retained 1.00, 0.96, 1.00 and the SNV-ANOVA50 pipeline retained 1.00, 0.98, 1.00 for plant category, origin, and year. The agreement between the two SNV pipelines shows that tolerance to multiplicative scaling arose from SNV, not from wavelength filtering.

The additive-offset test led to the same attribution. A random offset with standard deviation equal to 5% of each spectrum’s median intensity reduced the full-spectrum SVM to 0.92, 0.96, 0.96, while the full-spectrum plus SNV control retained 1.00, 0.96, 1.00 and the SNV-ANOVA50 pipeline retained 1.00, 0.98, 1.00. Thus, the comparison supports SNV as the protective step against uniform amplitude and baseline changes; the contribution of feature selection is not inferred from these perturbations.

Detector-channel displacement produced larger, task-specific losses. At a −2-index shift, plant-category balanced accuracy was 0.43, 0.94, and 0.26 for the full-spectrum, full-spectrum plus SNV, and SNV-ANOVA50 pipelines, respectively. At a +2-index shift, the corresponding origin values were 0.51, 0.97, and 0.67. Filtering to individual detector channels therefore increased vulnerability in some shift conditions and motivates calibration checks and window-based features.

## 3. Discussion

The LIBS system rapidly differentiated the measured pellet spectra by the assigned plant-category, origin, and growth-year labels. Plant category and growth year were nearly or completely separable across the tested pipelines, whereas geographical origin produced lower means and larger outer-fold variation. Because each class corresponds to one ground-and-sieved powder batch, these differences cannot be assigned uniquely to the nominal biological attribute; batch-specific soil, harvest, storage, or processing effects may also contribute. The results therefore establish technical within-batch separability, not general authentication performance.

The strong performance of the linear SVM indicates that a nonlinear classifier was unnecessary for separating the measured pellets. For plant category and growth year, a linear boundary matched or exceeded PCA-RBF-SVM and PLS-DA. Origin classification changed only modestly after SNV and ANOVA filtering. The new full-spectrum SNV control further shows that preprocessing and wavelength filtering must be interpreted separately: SNV explains tolerance to uniform scaling and offsets, whereas the 50-channel filter supplies dimensionality reduction and a way to examine recurrent wavelength regions.

Spectral compression showed that most detector channels were unnecessary for within-batch separation, but it did not identify a universal optimum. Fifty wavelengths represented 0.38% of the original channels and served as a common reference budget, while 10 channels were sufficient for the year task and 20 channels performed nearly as well as 50 for origin. The budget should therefore be selected for a defined task and confirmed using independent batches rather than transferred from the present compression curve as a fixed fingerprint size.

Feature stability provides information beyond mean predictive accuracy, but the ANOVA procedure is a univariate filter rather than a sparse embedded model. Variables repeatedly selected across 25 outer training folds are less dependent on one cross-validation split, yet adjacent detector channels may reflect the same broadened feature. A future model should use small wavelength windows, integrated peak areas, or ratios to stable reference lines and evaluate them with complete biological batches held out.

The conservative candidate matches do not justify chemical attribution. Plant genotype, soil parent material, fertilizer management, water availability, growth duration, storage, and processing can all affect LIBS measurements, and several of these factors were not recorded for the present materials. Matching was performed by comparing the selected wavelengths with tabulated NIST values and retaining only the nearest plausible candidate within 0.09 nm. No separate lamp-based wavelength calibration was performed for this dataset, and instrumental resolution is not proof of line identity; matrix-dependent blends remain possible. The retained offsets, 0.008–0.069 nm, do not indicate a common +0.12 nm correction. The table therefore reports tentative nearby lines, not elemental fingerprints or quantitative enrichment.

The perturbation tests separate normalization effects from wavelength-selection effects. The full-spectrum plus SNV control and the SNV-ANOVA50 pipeline showed similar resistance to uniform scaling and offsets, demonstrating that SNV was the principal protective step. In contrast, one- and two-channel shifts affected the filtered pipeline strongly in several task-direction combinations. This failure mode is not corrected by SNV and requires wavelength calibration or features defined over local windows.

This distinction has direct implications for instrumentation. Intensity variation can be reduced through normalization, laser-energy monitoring, replicate averaging, and internal reference features. Wavelength displacement requires routine calibration with known emission sources and feature definitions that tolerate local shifts. The asymmetric shift response observed here also argues against a single global correction selected from classification accuracy. Calibration should instead be established independently of class labels and verified across the spectral range so that apparent performance is not improved by aligning to the same samples used for model development.

The broader agricultural spectroscopy literature also indicates that preprocessing and validation must be matched to sample structure. Hyperspectral studies of Fritillaria, cat food, Curcumae Radix, goji berry, and bean seeds have used reconstruction models, classical chemometrics, nested validation, or deep networks to address different sources of variation [17,18,19,20,21]. Their common lesson is that high accuracy is meaningful only in relation to the materials, anomalies, morphologies, and acquisition conditions represented in evaluation. For the present LIBS experiment, the repeated nested design prevents preprocessing and tuning leakage at the pellet-spectrum level, but it cannot create biological variation that is absent from the sample collection.

The central limitation is the sampling hierarchy. Fifty pellets per class are technical preparation and measurement replicates from one ground-and-sieved powder batch, so class identity is completely confounded with batch identity. Pellet-level cross-validation can measure repeatability within these powders and can prevent computational leakage during tuning, but it cannot estimate generalization to new biological lots. Multiple independently sourced batches per class, recorded cultivar and production metadata, and batch-level holdout testing are required before the selected regions can support an authentication claim.

Within this boundary, the contribution is methodological rather than a new demonstration of population-level ginseng authentication. The study uses a separate LIBS acquisition experiment, isolates the information available from LIBS without data fusion, applies nested validation, quantifies filter stability across outer folds, and introduces perturbation controls that distinguish SNV effects from wavelength-selection effects. These results define testable procedures and failure modes for a future independently batched study.

## 4. Materials and Methods

### 4.1. Sample Preparation

(Eight ginseng materials representing three separate classification datasets were used. The plant-category dataset comprised Chinese, Korean, Japanese, and American ginseng; the origin dataset compared Changchun and Changbai Mountains materials; and the year dataset compared two- and four-year materials of the same plant category and geographical origin. The materials were obtained from the Zhongkepuguang Institute of Applied Science and Technology (Zhengzhou, China) and retail shops, following the sample-class design used in Ref. [1]. Slices were ground, passed through a 50-mesh sieve with a nominal aperture of approximately 300 μm, and pressed without binder into pellets approximately 30 mm in diameter and 2.5 mm thick using a hydraulic press at 25 MPa. Binder was unnecessary because the compressed powder formed self-supporting pellets for LIBS measurement; omitting it also avoided adding a foreign matrix component. Fifty pellets in each class were prepared from one ground-and-sieved powder batch and are technical replicates. The laboratory record contains no separate mixer type, mixing time, or mixing speed, so no additional mechanical homogenization step is claimed.

The class labels were those supplied with the source materials. Cultivar or genotype, exact collection coordinates, harvest year for datasets other than the explicit two- versus four-year comparison, soil and fertilizer history, storage duration, and processing history were not retained in the dataset, and origin and age labels were not independently re-verified. These missing metadata are potential confounders and restrict interpretation to the measured batches.

### 4.2. LIBS Acquisition

**Figure 7:**
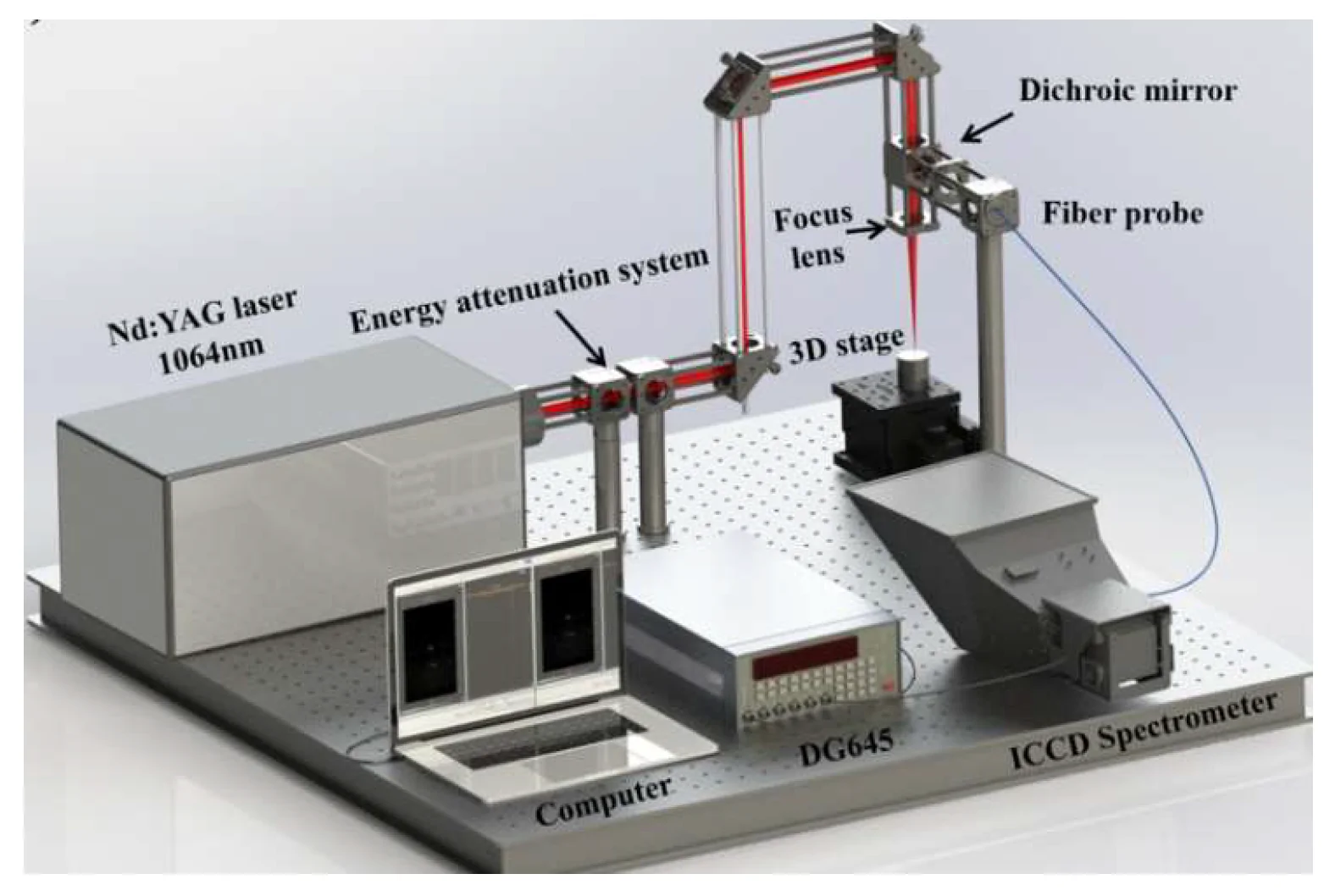
Schematic of the LIBS acquisition system. A Q-switched Nd:YAG laser was focused onto a binder-free ginseng pellet on a computer-controlled translation stage. Plasma emission was collected by fiber-coupled optics and recorded using seven synchronized linear-CCD spectrometer channels. Ninety spatially distributed acquisitions were averaged for each pellet.

Spectra were acquired using the ChemReveal LIBS (Shoreview, MN, USA) configuration employed in the earlier ginseng study [1] (Figure 7). The system contained a Q-switched Nd:YAG laser operated at 1064 nm and 2 Hz, with a pulse width of 7 ns and pulse energy of approximately 100 mJ. Plasma emission was recorded by seven synchronized linear-CCD channels of an Ocean Optics MX2500+ spectrometer (Orlando, FL, USA) covering 186.87–979.23 nm at a nominal resolution of 0.09 nm. Detector delay and gate width were 500 ns and 1 μs, respectively, and the focused laser spot was approximately 200 μm in diameter. These timing settings were adopted from the established system protocol and kept fixed; no delay or gate-width optimization series was performed in the present experiment. The nanosecond excitation conditions are conventional for qualitative LIBS emission measurements, but the experiment did not include the plasma diagnostics or response calibration required for quantitative elemental analysis [5,25].

A computer-controlled translation stage distributed 90 ablations across each pellet surface. The acquisition software stored their average as one 12,990-variable pellet spectrum; the individual-shot spectra and their coordinates were not retained. Consequently, the standard deviation of the 90 shots and a separate region-by-region reproducibility analysis cannot be reconstructed. Figure 1 instead shows variability among the 50 independently prepared pellet-level averages in each class.

### 4.3. Dataset Structure

No missing values, duplicate spectra, or constant wavelength variables were detected, and all tasks were class balanced. As shown in Table 3, the three datasets contain separate material sets. The origin data do not contain crossed growth-year labels, and the year data do not contain crossed origin labels; consequently, joint origin-year classification and cross-domain validation are not identifiable from these data. Combining them would instead create an eight-material batch classifier and would not disentangle origin from age.

**Table 3:** Structure of the three separate LIBS classification datasets.

Task	Classes	Spectra	Per Class	Variables
Plant category	4	200	50	12,990
Geographical origin	2	100	50	12,990
Growth year	2	100	50	12,990

### 4.4. Spectral Preprocessing and Classifiers

Four modeling strategies were compared. PLS-DA used PLSRegression with internal scaling and dummy-coded class targets [26]. The full-spectrum linear SVM applied StandardScaler across training spectra before fitting a linear SVM [27]. PCA-RBF-SVM centered the training data, retained 20 whitened principal components, and fitted an RBF-SVM. The filter-selected pipeline applied SNV to each spectrum, ranked all channels by an ANOVA F statistic computed only in the training fold, retained the top 50, applied StandardScaler, and fitted a linear SVM. SNV subtracts each spectrum’s mean and divides by its standard deviation [28]. Table 4 reports the search spaces and the modal choices across outer folds.

### 4.5. Nested Validation and Performance Metrics

High-dimensional predictors increase the risk of unstable selection and optimistic performance estimates when tuning and evaluation reuse the same observations [29,30,31]. Generalization within the measured powder batches was estimated using stratified five-fold outer cross-validation repeated five times, producing 25 held-out pellet-level evaluations per task. A stratified three-fold inner loop selected hyperparameters using balanced accuracy. Scaling, SNV, PCA, ANOVA filtering, and tuning were refitted using only each outer training fold. Reported standard deviations quantify variability across the 25 repeated outer-fold evaluations; these folds overlap in their training data and are not independent experimental replicates.

### 4.6. Feature Stability and Computational Stress Tests

For the SNV-ANOVA50 pipeline, channels appearing among the 50 selected variables in each outer training fold were counted. Selection frequency was the fraction of 25 outer folds containing a channel. Selected wavelengths were compared with tabulated NIST values, and a tentative candidate was retained only when the nearest plausible line differed by no more than 0.09 nm, equal to the nominal instrumental resolution [32]. Adjacent selected channels were examined as local neighborhoods, but no line deconvolution, lamp-based wavelength calibration, or standard-based identity confirmation was performed. Unmatched regions remain unassigned.

Compression experiments evaluated SNV-ANOVA linear SVMs using 10, 20, 50, 100, or 200 retained wavelengths. For the perturbation control, all linear SVMs used C = 1 so that pipeline structure, rather than retuning, defined the comparison: (i) StandardScaler plus full-spectrum SVM, (ii) SNV plus StandardScaler plus full-spectrum SVM, and (iii) SNV plus ANOVA50 plus StandardScaler plus SVM. Perturbations were applied only to held-out spectra. Multiplicative factors were drawn independently from Uniform (0.8, 1.2) for each spectrum. Additive offsets were drawn from a zero-mean normal distribution with standard deviation equal to 5% of that spectrum’s median intensity. Gaussian noise standard deviation was 1%, 3%, or 5% of that spectrum’s intensity standard deviation. Shifts moved the spectrum by one or two detector indices, and vacant boundary channels were filled with the nearest edge value.

## 5. Conclusions

This qualitative classification study shows that the measured ginseng powders contain compact LIBS signatures that support accurate pellet-level separation under controlled preparation and acquisition. Repeated nested cross-validation produced best mean balanced accuracies of 1.000 for plant category, 0.982 for geographical origin, and 1.000 for growth year. The standard deviations describe repeated outer-fold evaluations of technical replicates, not independent biological replication or uncertainty in elemental concentration. Geographical origin remained the least stable task and should receive priority in a future batch-structured experiment.

The spectra were highly compressible within the measured batches. The 50-channel SNV-ANOVA pipeline retained balanced accuracies of 1.000, 0.980, and 1.000 while using 0.38% of the variables, but 50 channels are presented as a common benchmark rather than a universal optimum. Conservative matching identified several tentative nearby Ba, Al, Ca, and Fe lines. These regions are hypotheses for targeted follow-up, not confirmed elemental identities or concentration differences.

The full-spectrum SNV control changed the interpretation of the perturbation analysis. Both SNV pipelines resisted uniform scaling and offsets, so this behavior is attributed primarily to normalization rather than to ANOVA filtering. Channel shifts caused larger and asymmetric losses, with the filter-selected pipeline particularly vulnerable in some conditions. Future measurements should combine routine wavelength calibration with window-based peak features and independent batch testing.

Each class was represented by one ground-and-sieved powder batch, making class identity inseparable from batch identity. The study therefore supports only technical within-batch separability. It does not establish that the selected wavelengths will transfer across cultivars, suppliers, harvest years, production environments, storage histories, acquisition days, or instruments. A valid authentication study should include multiple independently sourced batches per class and reserve entire batches for testing. Future LIBS experiments should also retain individual-shot spectra, test pellet regions explicitly, use a calibration lamp and certified reference materials, and measure plasma conditions when chemical quantification is intended. The present analysis provides a transparent computational protocol and identifies controls that should be carried into that study.

## Figures and Tables

**Table 1:** Repeated nested cross-validation performance (mean ± standard deviation across 25 repeated outer-fold evaluations; these evaluations are not independent biological replicates).

Task	Model	Balanced Accuracy	Macro-F1	MCC
Plant category	Full-spectrum linear SVM	1.000 ± 0.000	1.000 ± 0.000	1.000 ± 0.000
	PCA–RBF–SVM	0.993 ± 0.014	0.993 ± 0.014	0.991 ± 0.017
	PLS-DA	0.995 ± 0.010	0.995 ± 0.010	0.993 ± 0.013
	SNV-ANOVA50 SVM	0.999 ± 0.005	0.999 ± 0.005	0.999 ± 0.007
Geographical origin	Full-spectrum linear SVM	0.980 ± 0.029	0.980 ± 0.029	0.962 ± 0.054
	PCA–RBF–SVM	0.980 ± 0.025	0.980 ± 0.025	0.962 ± 0.048
	PLS-DA	0.968 ± 0.032	0.968 ± 0.032	0.939 ± 0.061
	SNV-ANOVA50 SVM	0.982 ± 0.024	0.982 ± 0.025	0.966 ± 0.047
Growth year	Full-spectrum linear SVM	1.000 ± 0.000	1.000 ± 0.000	1.000 ± 0.000
	PCA–RBF–SVM	0.994 ± 0.030	0.994 ± 0.031	0.989 ± 0.053
	PLS-DA	0.996 ± 0.014	0.996 ± 0.014	0.992 ± 0.026
	SNV-ANOVA50 SVM	1.000 ± 0.000	1.000 ± 0.000	1.000 ± 0.000

**Table 2:** Recurrently selected wavelengths with conservative tentative line matches restricted to an absolute difference of 0.09 nm.

Task	Selected (nm)	Frequency	Candidate Line (nm)	Difference (nm)
Plant category	455.466	0.76	Ba II 455.402	0.064
Geographical origin	309.314	1.00	Al I 309.283	0.031
	308.253	1.00	Al I 308.215	0.038
	396.221	0.96	Al I 396.152	0.069
Growth year	422.711	1.00	Ca I 422.672	0.039
	866.379	1.00	Fe I 866.371	0.008

**Table 4:** Preprocessing sequences, hyperparameter search spaces, and modal choices across the 25 outer training folds.

Pipeline	Preprocessing and Classifier	Search Space	Modal Inner-CV Choice by Task
PLS-DA	PLSRegression with internal scaling	*n* components = 2, 4, 6, 8, 10	Category: 6 (21/25); origin: 4 (13/25); year: 4 (22/25)
Full-spectrum linear SVM	StandardScaler → linear SVM	C = 0.01, 0.1, 1, 10	C = 0.01 for all tasks (25/25 each)
PCA-RBF-SVM	20-component whitened PCA → RBF-SVM	C = 0.1, 1, 10, 100; gamma = scale, 0.01, 0.1	Category: C 1, gamma 0.01 (18/25); origin: C 10, gamma 0.01 (14/25); year: C 1 or 10, gamma 0.01 (12/25 each)
SNV-ANOVA50 SVM	SNV → ANOVA top 50 → StandardScaler → linear SVM	C = 0.01, 0.1, 1, 10	Category: C 0.1 (15/25); origin: C 0.1 (14/25); year: C 0.01 (25/25)

## Data Availability

Data will be made available upon request.
